# Facilely Promoting the Concentration of Baicalin in Polylactic Acid Fiber for UV Shielding and Antibacterial Functions: A Customized and Sustainable Approach

**DOI:** 10.3390/ma17153734

**Published:** 2024-07-28

**Authors:** Yuyang Zhou, Peng Deng, Wei Chen

**Affiliations:** 1 National Engineering Laboratory for Modern Silk, National Textile and Apparel Council Key Laboratory of Natural Dyes, Jiangsu Engineering Research Center of Textile Dyeing and Printing for Energy Conservation, Discharge Reduction and Cleaner Production, College of Textile and Clothing Engineering, Soochow University, Suzhou 215123, China; dengpeng@alu.suda.edu.cn; 2 School of Art, Soochow University, Suzhou 215123, China; chenweisdyz@163.com

**Keywords:** polylactic acid, baicalin, sustainable finishing, ultraviolet protection, antibacterial

## Abstract

There is a significant trend towards the integration of natural substances with bio-polymers for fully bio-based functional composites. Polylactic acid is regarded as a promising biodegradable polymer for replacing synthetic polymers. Differing from the case of natural fiber, the incompatibility of polylactic acid with bio-based molecules prevents it from being used to fabricate high-quality sustainable composites. This work presents a simultaneous ultraviolet shielding and antibacterial finishing process of polylactic acid combined with bioactive baicalin and an eco-friendly ester, which is highlighted for (a) the lack of synthetic chemicals involved in such process, (b) adsorption enhancement achieved at a mild temperature, and (c) marginal color change on treated polylactic acid. A response surface methodology was adopted to analyze the impacts of various factors on the baicalin quantity in polylactic acid, and to optimize the treatment condition. The uptake ratio of baicalin in polylactic acid was drastically promoted from 8.5 mg/g to 21.1 mg/g using methyl cinnamate. The response surface methodology based on a central composite design experiment indicated that the usage of baicalin was the most significant factor followed by methyl cinnamate and temperature. After optimization, a very faint color depth of 1.2 was apparent, but UPF 50+ and 92% bacterial reduction could be achieved. In all, the success in strengthening of the functionalities of polylactic acid extends the applications of polylactic acid products.

## 1. Introduction

The worldwide exhaustion of fossil resources, along with concerns over microfiber pollution from synthetic textiles, have led the textile sector to seek eco-friendly alternatives. Polylactic acid (PLA), originating from natural sources like corn and sugar cane, has emerged as an ideal replacement for the prevalently used synthetic polyester fiber due to its crease resistance, better pill resistance than cotton, and superior hydrophilicity compared to that of PET [1,2]. The hydrophobic structure of PLA restricts its water absorption property, making it suitable for disperse dyeing at high temperature. Hence, most initial research on the coloration of PLA focused on the comparison between the chemical structure of disperse dyestuffs with their dyeing properties [2].

Nonetheless, several challenges hinder the further development of PLA in the textile domain. For example, though the elevated-temperature process enhances chemicals’ adsorption and diffusion into the PLA fiber through swelling and expansion, they concurrently compromise the fiber’s mechanical strength. Consequently, subsequent studies have concentrated on preserving PLA’s mechanical properties through alternative synthetic esters or techniques [3,4] to reduce the processing temperature. Secondly, the use of synthetic finishes undermines PLA’s sustainability. Pursuing eco-friendly chemical applications in textiles aligns with the Sustainable Development Goals (SDGs) and constitutes an encouraging research direction. Therefore, combining bio-based finishes with PLA is currently advocated. Lastly, PLA inherently lacks UV-absorbent structures, resulting in high UV transmission, which restricts its usage for outdoor or protective clothing applications.

Over the last few decades, considerable research has focused on integrating bio-extracts into PLA-based composites to boost their performance and mechanical attributes [5,6]. Techniques like melt spinning, coating, and ScCO_2_ processing are readily employed to integrate natural compounds with PLA for functional packaging films or composite materials [7,8]. However, when it comes to the production of PLA fibers, two key hurdles arise: (a) ensuring the consistent extrusion of fibers from the blend of PLA and bio-extracts and (b) preserving the bioactivity of these extracts during the high-temperature (>200 °C) melt spinning process. Therefore, post-processing treatments are often favored for PLA fibers, especially for cases involving bio-extracts.

Compared with synthetic fibers, natural dyeing and finishing techniques have been more extensively studied using hydrophilic natural fibers, such as cotton, silk, and wool, due to the strong affinity of bio-extracts to these fibers (occasionally with mordants) and the simplicity of water bath application methods. Our earlier work has demonstrated the successful imbuing of silk, wool, and cotton for multiple functionalities (including antimicrobial, antioxidant, UV protection, and flame retardancy) using bio-extracts with diverse structures [9,10,11]. The main challenge for PLA lies in the identification of suitable bio-extracts that can withstand high-temperature treatments without significant degradation and the promotion of the adsorption of bio-extracts onto PLA fibers.

To date, there are a few reports on the application of bio-extracts to PLA fibers. For example, *Rheum emodi,* having an anthraquinone structure similar to typical disperse dyes, displays a high affinity to PLA yarns [12]. More than ninety percent exhaustion of curcumin (diketone) was also achieved on PLA fibers. Flavonoids make up the majority of plant extracts; however their application to PLA fibers has not been sufficiently investigated. Beyond textiles, explorations of the adsorption of flavonoids on PLA have also extended its potential application to the medicinal or pharmaceutical fields (e.g., drug delivery, sustained-release implants). *Scutellaria baicalensis* Georgi, a fundamental herb in the annals of traditional Chinese medicine, has been utilized for centuries. Baicalin (5,6,7-trihydroxyflavone-7-*O*-glucuronide) is the key bioactive compound among the compounds of *Scutellaria baicalensis* Georgi. Extensive research has demonstrated baicalin’s broad spectrum of biological effects, encompassing antibacterial, antioxidative, anticancer, anti-inflammatory, and antiviral properties. Our earlier work integrated baicalin into silk, bestowing it with antimicrobial and antioxidant properties. Conversely, prior research established that disperse dyes, particularly azo dyes with solubility akin to PLA, and those rich in -NHR, -NHCOR, -OR functional groups over -NO_2_, -NH_2_, -OH, or -CN (where R represents -CH_3_, -(CH_2_)nCH_3_, or a phenyl group), demonstrate strong affinities for PLA [13]. Consequently, the adsorption of baicalin onto PLA fibers remains uncertain and might necessitate supplementary measures to boost its interaction.

Esters have emerged as potent enhancers in improving dye uptake thanks to their ability to stimulate fiber expansion and create broader pathways for dyes to infiltrate the PET fiber structure [14]. Cinnamic acid, a recognized natural aromatic carboxylic acid with roots in traditional Chinese medicine, can be derived from sources like Cinnamomum cassia (Chinese cinnamon) bark, fruits, whole grains, vegetables, honey, and more [15]. Methyl cinnamate (MC), a notable derivative of cinnamic acid, is valued for its low toxicity, ultraviolet absorption, and fungicidal properties [16,17]. It finds applications as a food preservative, cosmetic component, and pharmaceutical ingredient. However, its potential in augmenting adsorption quantity, particularly when combined with natural extracts and PLA, remains unexplored in the textile industry. It is hypothesized that MC could act as a softener, enabling better baicalin adsorption onto PLA fibers under heat. Furthermore, MC’s photo-induced trans–cis isomerization property suggests its potential to impart UV protection to PLA [18]. Driven by the relentless endeavor to develop entirely eco-friendly textiles, this paper introduces an innovative, sustainable approach to enhancing the adsorption of baicalin onto PLA using food-quality MC, thereby boosting its capacity for effective UV shielding and antibacterial properties. A Central Composite Design (CCD) experiment was employed to decipher the significance of individual factors, parameter interactions, and the optimal treatment conditions. Comprehensive evaluations were performed to gauge the UV protection and antibacterial efficacy of the modified PLA. This study endeavors to maximize the utilization of bio-extracts and their derivatives in creating a fully bio-based textile material, offering valuable insights for both academic researchers and industrial practitioners in advancing future research and product innovation.

## 2. Materials and Methods

### 2.1. Materials

Poly(lactic acid) fabric with a weight of 80 g/m^2^ was procured from Shenzhen Shengdefu Fiber Products Co., Ltd., based in Shenzhen, China. Baicalin, with a purity of 98%, was sourced from Xi’an Shouhe Biological Technology Co., Ltd., situated in Xi’an, China. Analytical-grade methyl cinnamate (MC) was supplied by both Shanghai Aladdin Bio-Chem Technology Co., Ltd. and Sinopharm Chemical Reagent Co., Ltd., both located in Shanghai, China. Additional chemicals, including acetic acid and sodium acetate, were of analytical grade and supplied by Jiangsu Qiangsheng Chemical Co., Ltd. in Changshu, China. All water utilized was ultrapure and obtained from a Millipore purification system (MilliporeSigma, Burlington, MA, USA).

### 2.2. Properties of Baicalin in Aqueous Solution

The assessment of baicalin’s calibration plot and stability preceded its implementation on PLA textiles. To gauge thermal stability, the samples were placed in an M-7-18P dyeing apparatus sourced from Wuxi Yangbo Printing and Dyeing Machinery Co., Ltd. (Wuxi, China). These were subjected to a simulated high-temperature dyeing procedure, commencing at 40 °C with a rate of 2 °C/min until reaching 110 °C, then maintained at that temperature with continuous agitation for 0 to 60 min. As for pH stability, adjustments were made using an HAc/NaAc buffer system.

### 2.3. Fabric Treatment

The treatment process involved submerging one gram of PLA fabric into a solution at a temperature of 40 °C. This was then gradually escalated to 110 °C at a rate of 2 °C per minute and maintained at that temperature for an interval of 40 min, utilizing a liquor ratio of 100:1. The MC was incorporated into the solution, and the bath’s pH was regulated through an acetate buffer to reach a value of 4.2. The baicalin concentration was varied from 0.5% to 15% based on the fabric’s weight. Upon completion of the treatment, the fabrics underwent triple rinsing with deionized water before being air-dried.

### 2.4. CCD Experiment

A central composite design (CCD) experiment, facilitated by Minitab 19’s trial edition (USA), was conducted as per the methodology outlined in Section 2.3, which pertains to fabric treatment. This investigation delved into the influence of three primary variables—baicalin concentration, MC concentration, and temperature—on the modified PLA’s adsorption capacity (Q expressed in mg/g). Both anticipated and observed values for these variables are presented in Table 1.

### 2.5. Measurement

#### 2.5.1. UV–Visible Spectroscopy and FTIR

UV–Vis absorption spectra and absorbance at the wavelengths of 317 nm for baicalin were acquired using a Shimadzu UV-1800 UV–Vis spectrophotometer, sourced from Kyoto, Japan. Fourier transform infrared (FTIR) spectroscopy was carried out on a NICOLET-iS5 FTIR spectrometer, a product of Thermo Fisher Scientific Inc., in the Waltham, MA, USA. This involved 32 scans with a resolution of 4 cm^−1^. Prior to the analysis, the fabric samples were meticulously prepared by cutting them into small sections, subsequently ground, combined with potassium bromide (KBr) powder, and finally compacted into thin disk pellets.

#### 2.5.2. UV-Protective and Antibacterial Performance

The assessment of the UPF value and the level of UV transmission was conducted utilizing a UV-2000F Labsphere UV transparency meter, a product of Labsphere Inc., situated in the North Sutton, NH, USA, adhering to the guidelines outlined in AS/NZS 4399: 2017 [19]. The antimicrobial effectiveness of the fabric against *E. coli* was evaluated by quantifying the decrease in bacterial colonies as per the standards outlined in GB/T 20944.3-2008 [20]. The *E. coli* (ATCC 8099) used in this study was obtained from the College of Life Science, Soochow University (Suzhou, China). For a comprehensive description, refer to our prior research works [10,21].

## 3. Results

### 3.1. Spectroscopy Analysis

#### 3.1.1. UV–Vis Adsorption Spectroscopy

**Figure 1:**
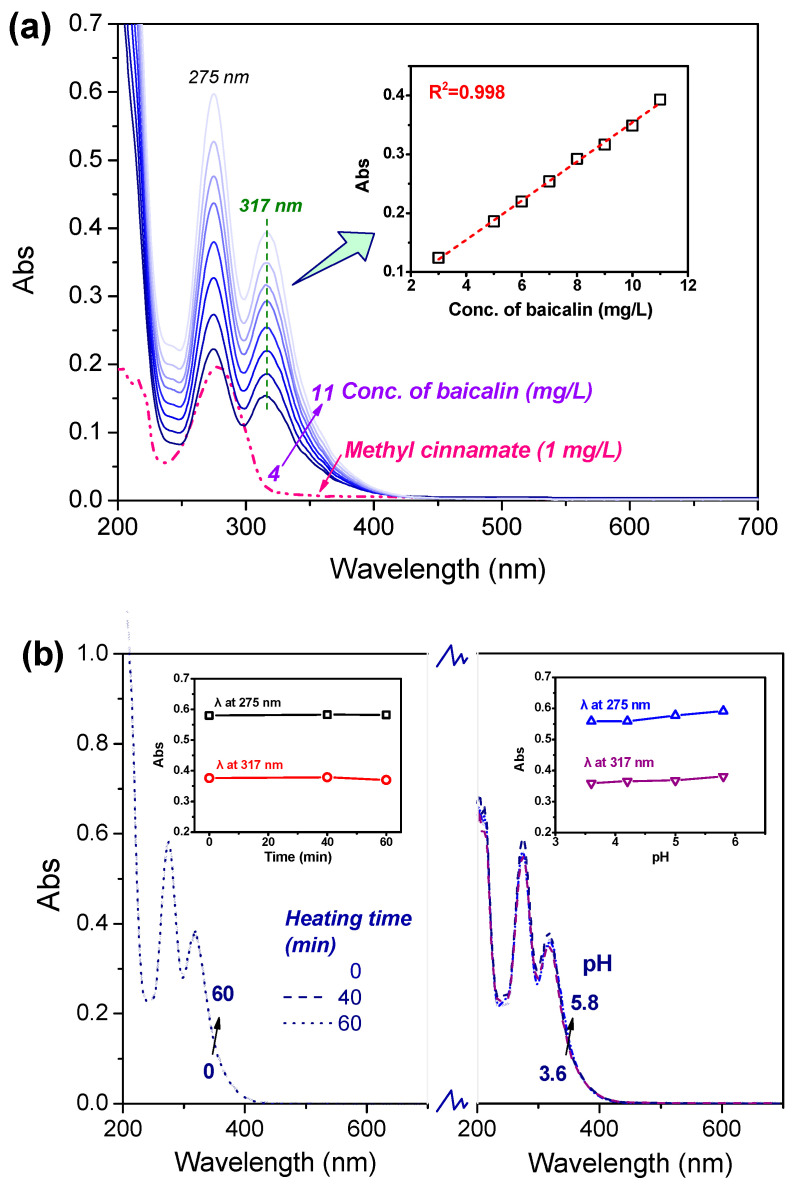
(**a**) UV–Vis spectra and calibration curve of baicalin in water solution, and (**b**) stability of baicalin under various heating periods and pH values. Note: The square, circle and triangle in the subfigure represent the Abs of corresponding spectrum at each maximum absorption wavelength.

As shown in Figure 1a, the UV–Vis absorption spectra of baicalin dissolved in water were analyzed across concentrations varying from 4 to 11 mg/L. Notably, two prominent peaks appeared at wavelengths of 275 nm and 317 nm, attributed to the A ring and the B/C rings present in baicalin’s molecular structure, respectively [10]. A calibration curve was constructed by linear regression analysis of the maximum absorbance at 317 nm against the serial concentrations, revealing a high R^2^ value near unity, signifying a strong correlation and suitability for quantifying baicalin concentrations in solution. Methyl cinnamate demonstrated significant absorption within the UV part of its spectrum, validating its UV absorption properties. Crucially, neither baicalin nor methyl cinnamate displayed light absorption within the visible-light spectrum (400–700 nm). This characteristic is advantageous for their use in PLA, as it minimally impacts coloration, making the finish esthetically neutral. Consequently, any coloration requirements would remain unaffected by this treatment.

The preservation of baicalin’s integrity in aqueous solutions throughout PLA processing, with emphasis on pH conditions and temperature influences, is crucial for maintaining the efficacy of the end product. Two identical samples of baicalin (11 mg/L) dissolved in water were prepared to examine its stability across different pH levels and exposure to heat. As depicted in Figure 1b, the UV–Vis absorption profiles remain superimposed from 0 to 60 min of heating, and notably, the absorbance at both λ_275 nm_ and λ_317 nm_ exhibit minimal alteration, indicating the remarkable thermal stability of baicalin. A slight upsurge in absorbance can be observed in the spectral range of baicalin’s aqueous solution between pH 3.6 and 5.8, which can be attributed to enhanced solubility resulting from the deprotonation of hydroxyl groups. Overall, baicalin displays a stable behavior throughout the heating process under these varying pH conditions.

#### 3.1.2. FTIR

**Figure 2:**
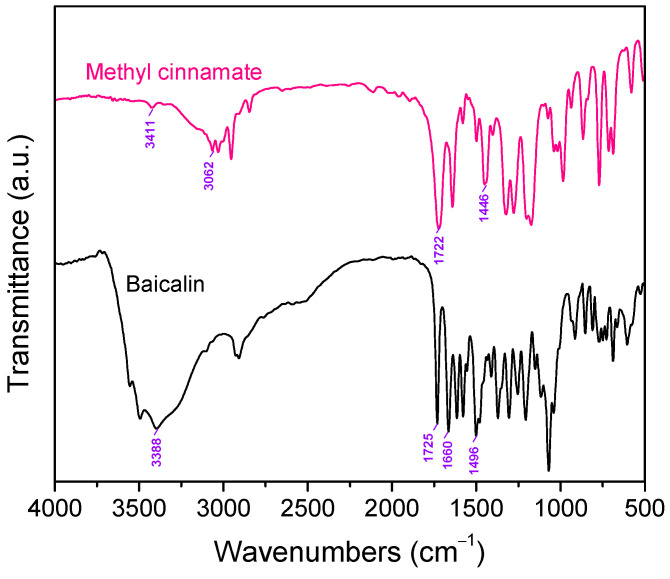
FTIR spectra of methyl cinnamate and baicalin.

The FTIR spectra of methyl cinnamate and baicalin are depicted in Figure 2. The featured peaks of baicalin are present at 3388 cm^−1^ (OH stretching), 1725 and 1660 cm^−1^ (C=O stretching), and 1496 cm^−1^ (C=C stretching). The angular deformation of C=CH of the aromatic compounds appears between 650 and 1000 cm^−1^. The characteristic peaks of methyl cinnamate are 3411 cm^−1^ for O-H stretching, 3062 cm^−1^ for C-H aromatic stretching, 1722 cm^−1^ for C=O stretching, and 1446 cm^−1^ for C=C aromatic stretching.

### 3.2. Methyl-Cinnamate-Assisted Finishing

**Figure 3:**
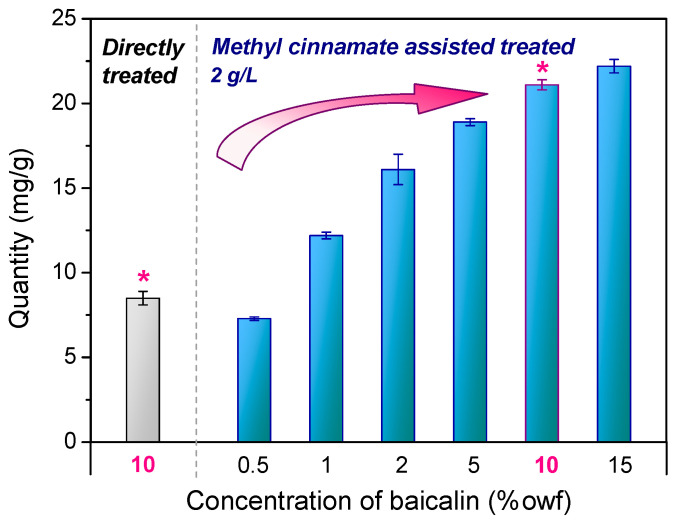
Adsorption quantity of baicalin on PLA with and without methyl cinnamate. Note: The asterisks represent the same baicalin concentration usage for comparison.

To explore the influence of methyl cinnamate on the adsorption of baicalin onto PLA, two methods were employed: direct baicalin treatment and methyl-cinnamate-assisted baicalin treatment. As depicted in Figure 3, the asterisks indicate two samples using the same concentration (10% owf) of baicalin with and without ester for easy comparison. Only 8.5 mg/g of baicalin (with an initial concentration of 10% owf) adhered to the PLA devoid of esters. In stark contrast, the presence of methyl cinnamate facilitated the attachment of 21.1 mg/g of baicalin, marking a significant increase of 2.5 times. This augmentation is attributed to the ‘molecular lubricant’ role of esters, which, upon heating, distend the space amid polymer chains, boosting baicalin’s mobility within the amorphous regions of the PLA fibers. The enhanced solubility of baicalin in the company of esters also plays a part, dissolving the baicalein aggregates into individual molecules, facilitating adsorption. Furthermore, the quantity of baicalin absorbed onto PLA can be regulated by varying the concentration of baicalein, catering to diverse application requirements when methyl cinnamate is present.

### 3.3. CCD Experiment

An extensive CCD study was conducted to delve deeper into the factorial influences on the quantity of baicalin absorbed by PLA compared with a single-factor experiment. A paramount equation was derived to illustrate the connection between treatment parameters and baicalin adsorption. Employing a response surface methodology facilitated the comprehensive analysis of the collected data. Described in Table 1, the factors under investigation were x_1_. baicalin concentration (1.3~9.7% owf), x_2_. MC concentration (0.49~3.01 g/L), and x_3_. temperature (91.6~108.4 _°_C). Therefore, the formula was formed as follows:

Q = A + Bx_1_ + Cx_2_ + Dx_3_ + Ex_1_x_2_ + Fx_1_x_3_ + Gx_2_x_3_ + Hx_1_^2^ + Ix_2_^2^ + Jx_3_^2^

where x_1_, x_2_, and x_3_ are variables; A is a constant; B, C, and D are the linear coefficients; E, F, and G are the interactive coefficients; H, I, and J are the quadratic coefficients.

The actual equation generated from modeling is shown below:

Q = −342.2 + 2.18 × Conc.(Bai) − 9.80 × Conc.(MC) + 7.15 × Temp. − 0.2840 × Conc.(Bai) × Conc.(Bai) − 0.877 × Conc.(MC) × Conc.(MC) − 0.03711 × Temp. × Temp. + 0.980 × Conc.(Bai) × Conc.(MC) + 0.0140 × Conc.(Bai) × Temp. + 0.1467 × Conc.(MC) × Temp.

Table 2 exhibits the factorial table and the computed versus actual adsorption capacity (mg/g). As detailed in Table 3, the model’s F value (227.32) evidences its substantial relevance. The linear terms (B and C) and the interaction term (E) exhibit insignificant *p*-values (less than 0.001), suggesting their profound influence on baicalin adsorption onto PLA. The ‘lack-of-fit’ *p*-value (0.806) exceeds the threshold of 0.05, indicating non-significance. The predicted R-Sq (98.4%) closely matches the adjusted R-Sq (99.1%), reflecting strong consistency between the predicted and experimental results. The high R^2^ value of 99.5% confirms the model’s exceptional suitability for predictive purposes.

**Figure 4:**
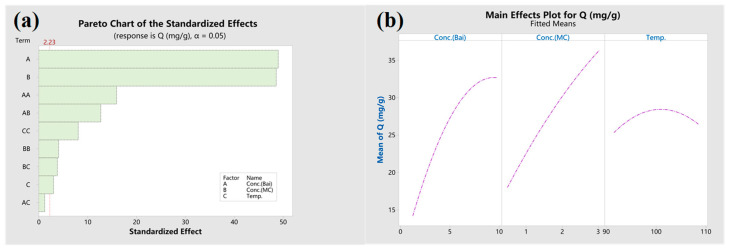
(**a**) Pareto chart and (**b**) main effects plot for PLA dyeing with baicalin/methyl cinnamate.

Plots for main effects and interactions facilitate the comprehension of variable significance and their interplays [22]. Pareto diagrams serve to illustrate the magnitudes of effects. As depicted in Figure 4a’s Pareto chart and the main effect plot in Figure 4b, baicalin and MC concentrations emerge as prominent variables, followed by temperature, a finding that aligns with their respective *p*-values in Table 3. Notable interactions occur between Conc.(Bai)×Conc.(MC) and Conc.(MC)×Temp. The interaction between Conc.(Bai)×Conc.(MC) suggests that the presence of MC boosts baicalin’s solubility and mobility, thus augmenting the final adsorption amount. Elevated temperatures further augment fiber expansion in conjunction with MC. Conversely, there is minimal interaction between Conc.(Bai)×Temp. influencing the absorption quantity.

**Figure 5:**
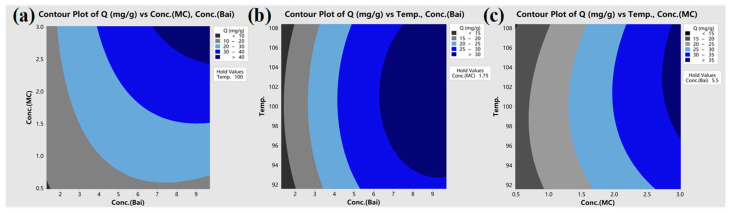
Contour plot of baicalin adsorption quantity as a function of each two factors: (**a**) Conc. of baicalin and MC, (**b**) Conc. of baicalin and temperature, and (**c**) Conc. of MC and temperature. Note: Conc. and MC are short for concentration and methyl cinnamate, respectively.

**Figure 6:**
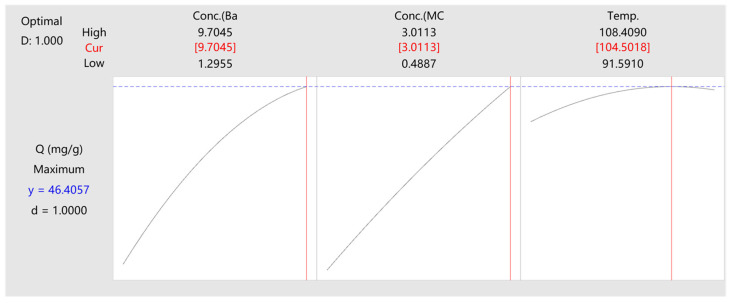
Optimization for baicalin adsorption quantity.

A contour diagram (Figure 5) is presented to illustrate the influence of parameters based on the quadratic model. The area depicted in deep blue (Figure 5a) signifies an elevated yield, situated in the upper right corner, indicating improved performance with increased baicalin or MC levels. Surprisingly, Figure 5b,c show that the maximum yield does not correspond to the maximum temperature. This implies that surpassing a certain temperature negatively impacts baicalin loading. The explanation lies in the fact that when temperature increases from 90 °C, the expansion of PLA, fostered by MC, intensifies, along with the solubility and mobility of baicalin, promoting its infiltration into the polymer chain gaps. PLA, more susceptible to heat than standard PET, particularly with MC-assisted fiber expansion, undergoes irreversible over-expansion at high temperatures, like at 108 °C, as depicted in the figure. This expansion creates unstable gaps between PLA chains, causing baicalin molecules to detach during rinsing. Hence, temperature selection must be cautious. Ultimately, an ideal processing condition is derived (baicalin concentration: 0.97 g/L, MC concentration: 3.0 g/L, temperature: 104.5 °C), predicting a maximum theoretical Q of 46.41 mg/g for PLA (Figure 6).

### 3.4. Functionality

**Figure 7:**
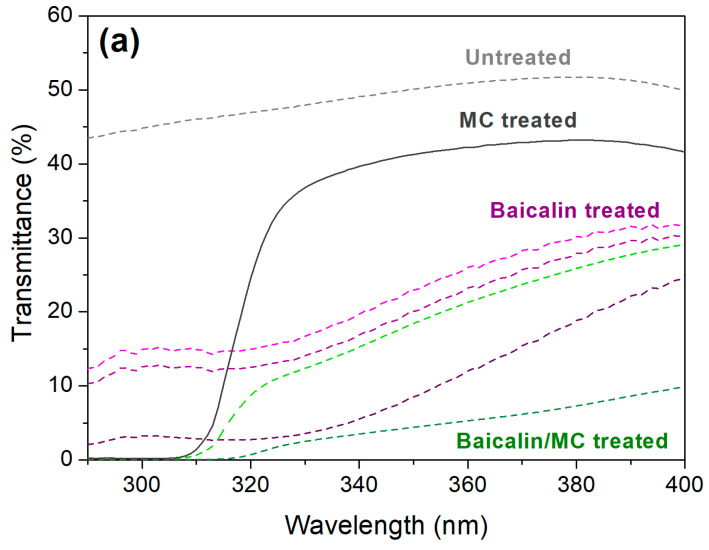

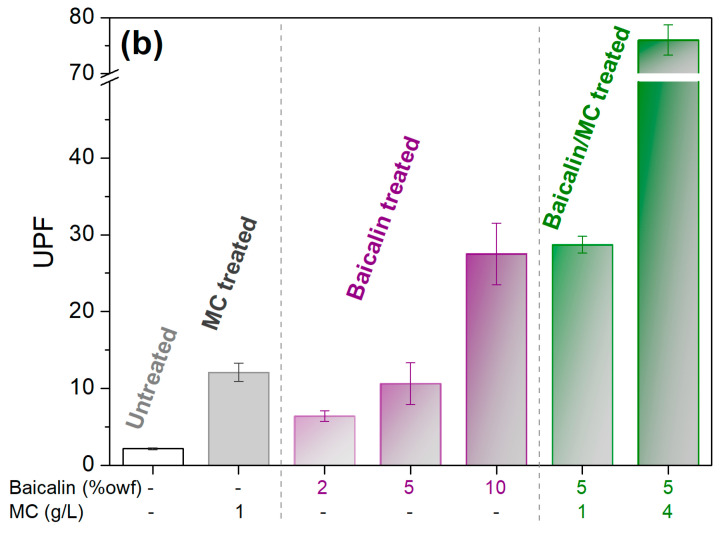
(**a**) UV transmittance and (**b**) UPF of PLA sample.

As illustrated in Figure 7, untreated PLA exhibits substantial transparency to both UVB (280 to 320 nm) and UVA (320 to 400 nm) wavelengths, leading to a low ultraviolet protection factor (UPF). This vulnerability to UV radiation can be attributed to PLA’s aliphatic configuration, which lacks adequate UV absorption properties. On the other hand, myricetin (MC) displays potent UVB absorbance capabilities (as evident from the UV–Vis absorption spectra in Figure 1a) due to its trans–cis isomerization under UV exposure [23], thereby escalating PLA’s UPF rating beyond 10. Baicalin, at a concentration of 10% owf, significantly impedes UV transmission through the PLA fabric, particularly in the UVA region, boosting the UPF to 27. The UVA absorption property of baicalin is primarily linked to its cinnamoyl moiety [24]. Nevertheless, baicalin alone demonstrates less efficacy in curbing UVB transmission than MC, a discrepancy echoed in their respective UV–Vis absorption spectra in Figure 1a. When combined, baicalin and MC jointly diminish UV transmission across both UVB and UVA bands, significantly amplifying the UPF. As per the AS/NZS 4399: 2017 standard [19], the PLA material treated with 5% owf baicalin and 4 g/L MC qualifies as a UPF 50+ product. This superior UV protection is largely owing to the high baicalin content of PLA in conjunction with MC’s residual presence on the material.

**Figure 8:**
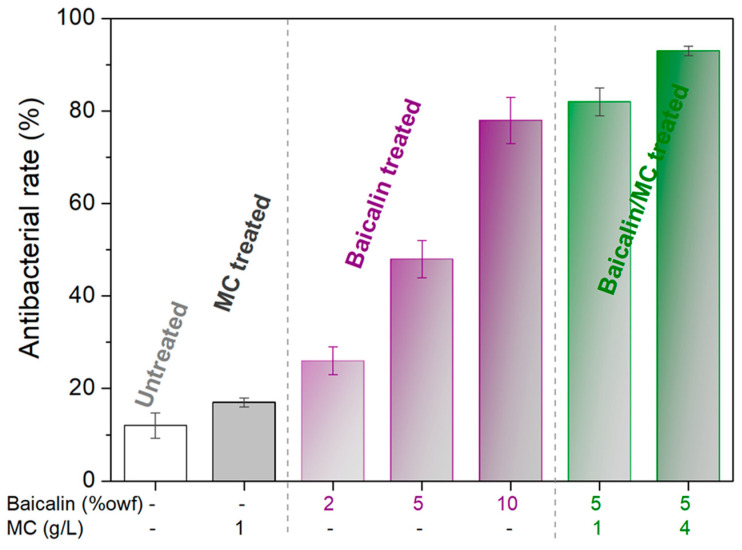
Bacterial reduction in PLA samples.

Textiles often undergo antibacterial treatments to avert disease transmission and minimize the risk of infection from injuries. These treatments also control odor formation from sweat, stains, and soil on fabrics, as well as hinder fabric degradation due to mold growth. As per previous research, baicalin is believed to hinder fungal growth possibly through disrupting the cellular wall and membrane integrity. As depicted in Figure 8, the untreated PLA fabric demonstrates weak antibacterial properties, demonstrating only a 16% inhibition rate against *E. coli*. Conversely, the baicalin-treated PLA fabrics display significant antibacterial efficacy against *E. coli*, albeit with an inhibition rate below 80%. A substantial improvement is evident when baicalin is combined with MC treatment. The PLA fabric treated with 5% owf of baicalin and 4 g/L of MC exhibits a 92% antibacterial rate, significantly outperforming the 10% owf baicalin-treated PLA.

## 4. Conclusions

This study investigated the application of baicalin in conjunction with MC for boosting adsorption rates, thereby endowing PLA with enhanced ultraviolet protection and antibacterial characteristics. Through a CCD experimental design, this study delved into the factorial influences and identified the optimum conditions. The findings reveal that the presence of MC increases baicalin’s adsorption onto PLA from 8.5 mg/g to 21.1 mg/g. In the CCD analysis, the significance of factors ranked as follows: baicalin concentration > MC concentration > temperature. Under the optimal parameters (0.97 g/L baicalin, 3.0 g/L MC, and a temperature of 104.5 °C), a calculated maximum adsorption capacity of 46.4 mg/g is achievable. The treated PLA exhibited superior UV defense with a UPF 50+ rating and demonstrated 92% antibacterial efficacy. This research validates the effectiveness of MC in augmenting baicalin’s adsorption onto PLA while imparting beneficial properties. Overall, although large enhancement in baicalin loads was achieved on PLA using MC, further detailed investigations on the property of as-prepared PLA compared with conventionally fabricated PLA are required. Future work is envisaged to enhance longevity and control release, integrating sustainable methodologies to further enhance the eco-friendliness of the process.

## Figures and Tables

**Table 1:** Matrix for variables and levels.

Variables	Levels				
	−α	−1	0	1	α
A: Conc.(Bai) (% owf)	1.3	3	5.5	8	9.7
B: Conc.(MC) (g/L)	0.49	1	1.75	2.5	3.01
C: Temp. (°C)	91.6	95	100	105	108.4

**Table 2:** Experimental runs and responses.

Run	Conc.(Bai) (% owf)	Conc.(MC) (g/L)	Temp. (°C)	Quantity (mg/g)	
				Predicted	Actual
1	3	1	95	16.79	16.68
2	8	1	95	23.63	23.21
3	3	2.5	95	22.79	22.48
4	8	2.5	95	36.98	36.64
5	3	1	105	16.01	15.90
6	8	1	105	23.55	23.41
7	3	2.5	105	24.21	24.17
8	8	2.5	105	39.10	38.75
9	1.3	1.75	100	14.42	14.54
10	9.7	1.75	100	32.70	33.22
11	5.5	0.49	100	18.12	18.37
12	5.5	3.01	100	36.24	36.64
13	5.5	1.75	91.6	25.39	25.87
14	5.5	1.75	108.4	26.52	26.68
15	5.5	1.75	100	28.58	28.39
16	5.5	1.75	100	28.58	29.22
17	5.5	1.75	100	28.58	27.99
18	5.5	1.75	100	28.58	29.59
19	5.5	1.75	100	28.58	28.68
20	5.5	1.75	100	28.58	27.49

**Table 3:** Model analysis.

Source	DF	Adj SS	Adj MS	F-Value	*p*-Value
Model	9	885.146	98.350	227.32	<0.001
Linear	3	800.961	266.987	617.11	<0.001
Conc.(Bai)	1	403.018	403.018	931.53	<0.001
Conc.(MC)	1	396.407	396.407	916.24	<0.001
Temp.	1	1.536	1.536	3.55	0.089
Square	3	54.522	18.174	42.01	<0.001
Conc.(Bai)×Conc.(Bai)	1	45.390	45.390	104.91	<0.001
Conc.(MC)×Conc.(MC)	1	3.510	3.510	8.11	0.017
Temp.×Temp.	1	12.402	12.402	28.67	<0.001
2-Way Interaction	3	29.663	9.888	22.85	<0.001
Conc.(Bai)×Conc.(MC)	1	26.998	26.998	62.40	<0.001
Conc.(Bai)×Temp.	1	0.246	0.246	0.57	0.468
Conc.(MC)×Temp.	1	2.420	2.420	5.59	0.040
Error	10	4.326	0.433		
Lack-of-Fit	5	1.322	0.264	0.44	0.806
Pure Error	5	3.004	0.601		
Total	9	885.146	98.350	227.32	<0.001

## Data Availability

No new data were created or analyzed in this study.
